# Long-term tremor therapy for Parkinson and essential tremor with sensor-guided botulinum toxin type A injections

**DOI:** 10.1371/journal.pone.0178670

**Published:** 2017-06-06

**Authors:** Olivia Samotus, Jack Lee, Mandar Jog

**Affiliations:** 1 London Health Sciences Centre – Lawson Health Research Institute, Department of Clinical Neurological Sciences, London, Ontario, Canada; 2 University of Western, Schulich School of Medicine and Dentistry, London, Ontario, Canada; University of Toronto, CANADA

## Abstract

**Objective:**

Current pharmacological agents used to treat Parkinson disease (PD) tremor and essential tremor (ET) provide suboptimal benefit and are commonly associated with significant adverse effects. Botulinum toxin type A (BoNT-A) has been shown to be effective for wrist tremor though functionally bothersome muscle weakness frequently occurs. This is the longest study to date demonstrating that BoNT-A therapy coupled with kinematic guidance can provide efficacious outcomes for upper limb tremor with minimized unwanted weakness.

**Methods:**

A total of 28 PD and 24 ET participants with bothersome, disabling tremor, received six serial BoNT-A treatments every 16 weeks starting at week 0 with a follow-up visit 6 weeks following a treatment, totaling 96 weeks. Clinical scales, including Fahn-Tolosa-Marin tremor rating scale (FTM), and sensor-based tremor assessments were conducted at each visit. Kinematics was utilized to identify which arm muscles contributed to the tremulous movements and the experienced injector used clinical expertise in determining BoNT-A dosages.

**Results:**

Following BoNT-A treatment, clinical ratings of tremor severity and functional ability (FTM) showed significant improvements following the first treatment which was maintained up to week 96 in PD and ET. Kinematics detected a significant reduction in PD and ET tremor amplitudes by 70% and 76% over the treatment course, respectively. By objectively distinguishing tremulous muscles and tremor severity, adverse effects were limited to mild perceived weakness by participants in injected muscles during follow-ups. Following the fourth treatment, BoNT-A dosages in flexor and extensor wrist muscles and biceps were reduced for those experiencing residual weakness which ultimately did not interfere with tremor relief or arm function.

**Conclusions:**

Kinematics is an objective method that can aid clinicians in assessing and determining optimal BoNT-A parameters to alleviate both PD and ET tremor. BoNT-A injections are tolerable and effective when focal therapy regimens are determined and optimized kinematically over a long-term.

## Introduction

Tremor greatly affects Parkinson disease (PD) patients during rest and is a functional interference and social embarrassment for essential tremor (ET) patients; hence many ultimately seek therapy. Unfortunately, the treatment of tremor is a significant unmet need as traditional pharmacotherapy, such as levodopa and dopamine agonists for PD tremor and beta-blockers and anticonvulsants for ET, produces suboptimal benefit and is frequently associated with significant adverse events, such as fatigue, cognitive and neuropsychiatric side effects [1–3]. Surgical interventions such as deep brain stimulation and thalamotomy are effective for treating oral drug resistant ET and PD tremor, but are highly invasive procedures and further studies are required to establish guidelines for optimizing device programming and decreasing frequent side-effects [4–7]. Although tremor can be generalized, upper limb tremor is commonly more disabling and hence targeted therapy such as botulinum toxin type A (BoNT-A) injections must be considered.

In a limited number of studies, BoNT-A injections for treating upper limb ET and PD tremor showed a modest response in reducing tremor amplitudes, though functional benefit was limited by potential hand muscle weakness [8–12]. Thus, further studies are needed to refine injection guidelines, importantly selection of tremulous muscles and optimal BoNT-A dosages. Visual assessment of tremor is highly subjective and it can be challenging even for experienced clinicians to accurately determine which joints are affected by tremor and severity at each joint due to the high variability of tremor in multiple joints throughout the day [13]. This complex visual assessment would hinder a clinician from optimizing injection parameters if BoNT-A was tried. Accelerometric sensors and validated rating scales can provide an overall arm tremor severity score but have not been reliably used for BoNT-A dosing.^12^ Previously described by Rahimi and colleagues, multi-sensor kinematic technology is an effective technique for personalizing and optimizing BoNT-A therapy thereby ultimately reducing tremor amplitudes and improving quality of life (QoL) and clinical arm functionality in ET and PD patients [14–16]. By targeting muscles that contribute to overall tremor, the likelihood of muscle weakness in these patients was minimized over three serial treatments [14,15]. The current study reports the 2 year longitudinal results on the efficacy and tolerability of kinematically-guided, individualized BoNT-A injections for management of debilitating upper limb ET and PD tremors.

## Methods

**Fig 1:**
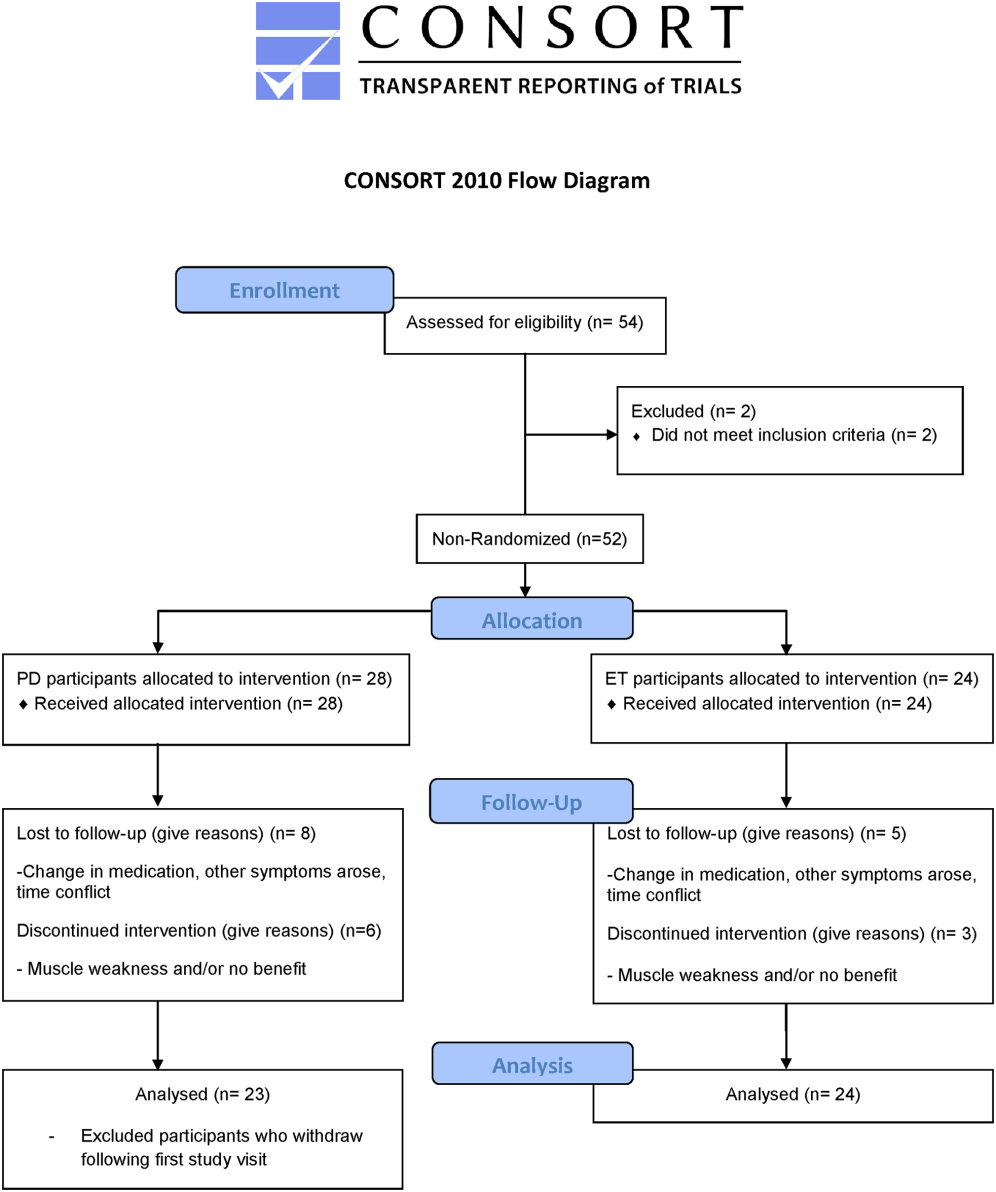
Study design, follow-up and analysis of participant datasets in the form of a CONSORT flow diagram.

This clinical phase II pilot study protocol, previously described in Samotus et al 2016 and in Rahimi et al in 2015, was approved by the Western University Health Sciences Research Ethics Board as an open label, single-centre, single-injector study (Health Canada CTA# 178589) on March 28, 2012 [14,15]. Full study protocol along with the TREND statement checklist is listed in S1 and S2 Files. First participant’s first visit and last participant’s last visit occurred in April 2012 and May 2015, respectively. The study and all related trials for this intervention are registered on the clinicaltrials.gov registry (ClinicalTrials.gov Identifier: NCT02427646). The ethics committee provided full board approval for this study protocol and consent procedure was approved as required in the consent documentation checklist, submitted with the full study protocol. Registration with a clinical trial registry was not a requirement for ethics approval to perform the study at this Canadian institution. The authors confirm that all ongoing and related trials for this drug/intervention are registered. Fig 1 outlines the study design and analysis in a CONSORT flowchart.

A convenience sample of 28 PD participants and 24 ET participants were recruited for this open-label, pilot study with no randomization from the London Movement Disorders Centre (LondonMDC) in London, Ontario, Canada. The power calculation included in the ethics study protocol (see supplementary material) suggested a target sample size of 35 ET and 35 PD participants; however this calculation was based on literature that did not involve objective analysis for guiding BoNT-A injections. Participants attended a total of 13 study visits over a 96-week duration; BoNT-A (incobotulinumtoxinA; Xeomin^®^) treatments were administered every 16 weeks on weeks 0, 16, 32, 48, 64, 80, totaling six treatments, and a follow-up visit occurred six weeks post-treatment on weeks 6, 22, 38, 54, 70, 86, with the final follow-up visit at week 96. Clinical and kinematic assessments were performed at all visits and peak effects of BoNT-A were measured six weeks post-treatment [9,14,15].

### Eligibility criteria

Participants recruited met similar inclusion/exclusion criteria as described in Samotus et al 2016 and in Rahimi et al 2015 [14,15]. Inclusion criteria were: female and male participants with upper limb tremor as their most bothersome and primary symptom for a minimum of two years, must be BoNT-A naïve and on stable medication management for a minimum of 6 months prior to enrolment. ET participants included in this study were diagnosed with ET based on Tremor Investigation Group (TRIG) criteria and PD participants were defined as idiopathic PD by UK Brain Bank Criteria (Hoehn-Yahr stages 1–3). Exclusion criteria were: those with history of stroke, contradictions to BoNT-A drug monograph [17], pregnancy, and existing pharmacological therapy with tremor-inducing side effects (lithium, valproate). No medications were added, withheld or adjusted during the entire study. Only one of the upper limbs, deemed by the participant to be most bothersome, was injected in all participants, as some participants presented with bilateral tremor.

### Types of clinical outcome measures

Severity of tremor in PD and ET individuals was reported using established tremor rating scales such as the Fahn-Tolosa-Marin (FTM) tremor rating scale and the Unified Parkinson’s disease rating scale (UPDRS) motor items, particularly items 20 and 21 representing rest and action (in the posture position) tremors, respectively, at each study visit [18,19]. All UPDRS assessments were conducted by the same clinician who was blinded to the prior scores and to the treatment parameters. The FTM scale assesses tremor severity (part A) during rest, posture and action positions, ability to write and pour liquids (part B), and functional disability caused by tremor (part C). Quality of life (QoL) measures in ET individuals were reported using the quality of life for essential tremor questionnaire (QUEST), 30-items assessing psychosocial, communication, and physical activities on a scale ranging from 0: never, 1: rarely, 2: sometimes, 3: frequently, and 4: always [20]. Overall QoL and health measures for PD participants were self-reported using a visual analogue scale (VAS) ranging from 0: no QoL/health to 100: perfect QoL/health. Side effect(s) to BoNT-A injections, mainly muscle weakness, was monitored using a Baseline^®^ hydraulic hand dynamometer (Item#:12–0240, White Plains, NY) to measure maximal grip strength and a Likert style participant-ranked scale (ranging from 0: no weakness, 1: mild, 2: moderate, 3: marked and 4: severe weakness with functional loss in injected muscles) to assess perceived muscle weakness reported by all participants at each visit. Manual muscle testing (MMT) was used to assess finger flexor/extensor muscles where scores ranged from 0,1,2-,2,2+,3-,3,3+,4-,4,4+,5 with a score of 3+ or higher representing the ability to hold muscle position against gravity and resist slight pressure [21,22].

### Kinematic analysis of tremor arm

Participants identified which tremor arm was most bothersome which was then selected for kinematic measurements and treatment. Participants were assessed at the same time of day to reduce variability in tremor severity. Presence and severity of upper limb tremor was captured while participants performed a series of six scripted tasks each held for 20 seconds over three trials, as previously described [14,15]: two rest positions with the forearm supported in lap (“rest-1”) or supported on a board (“rest-2”), two postural positions with the arms pronated outstretched with palms facing downwards (“posture-1”) or with arms semi-supinated outstretched with palms facing each other (“posture-2”), and two weight-bearing tasks where participants held an empty cup (“load-1”) or held a cup with a 1-lb weight (“load-2”) as previously illustrated in Samotus et al [14].

All participants were assessed while “ON” their medication as this was identified to be most representative of the pharmacologically unresponsive state of tremor. Motion sensor devices, goniometers (Biometrics Ltd., SG150) placed over the wrist, elbow and shoulder joints, and a torsiometer (Biometrics Ltd., Q150) placed along the forearm, captured tremor by angular amplitude as previously described [14,15]. Wrist tremor was measured in multiple degrees of freedom (DOFs), flexion-extension (F/E) and radial-ulnar (R/U) deviations where the third DOF was pronation-supination (P/S) movements about the wrist. Elbow tremor was captured in F/E DOF and shoulder tremor was segmented into two DOFs, F/E and abduction-adduction (A/A) deviations. Sensor data was collected in real-time by TeleMyo^™^ 2400T G2 at 1500Hz and transmitted to a computer running MyoResearch XP Version 1.08.09. There was no cross-talk between sensors, thus movement captured from each joint was captured on individual channels.

Tremor features were extracted from the angular signal data using custom-written software in MatLab^®^ (Version 2011a) [14–16]. For each participant, mean total tremor amplitude at each joint was displayed as angular root mean squared (RMS) amplitude degrees for each scripted task over three trials. RMS units is a standard method of analyzing tremor severity and combining data from different degrees of freedom from the same origin [14–16]. Wrist and shoulder tremors detected during each task were further segmented into the amount of tremor present in each DOF that contributes to the overall total tremor at that joint, represented as a percentage.

### BoNT-A intervention approach

For each arm joint, a single experienced injector reviewed each participant’s baseline kinematic tremor readout. This kinematic assessment replaced the subjective visual assessment and an example of how the selection and determination of BoNT-A parameters have been previously published in Samotus et al [14]. Using best clinical judgement, the injector allocated a BoNT-A dose based one of the six tasks which produced the highest total tremor amplitude at that joint. The selected dosage for the wrist and shoulder joints were divided based on the amount/percentage of tremor in each DOF the joint moves in. A total of seven wrist muscles and four shoulder muscles were targeted: flexor carpi radialis (FCR), flexor carpi ulnaris (FCU), extensor carpi radialis longus (ECR), extensor carpi ulnaris (ECU), pronator teres (PT), pronator quadratus (PQ), supinator, pectoralis major, teres major, deltoid and supraspinatus. The allocated dosage for the elbow joint was divided based on the number of muscles targeted, biceps brachii and triceps. Muscle injection sites were selected based on the known anatomical contribution to the movement of each joint. Hence, BoNT-A dosages were distributed to the appropriate muscle groups using kinematics [14,15]. Optimization of BoNT-A risk/benefit parameters, as previously described by Samotus et al, required the clinician to compare the kinematic changes in tremor from pre-injection to post-injection visits along with participant feedback regarding efficacy and weakness [14].

### Statistical analysis

Clinical rating scale scores were represented as mean and standard deviations of the population for each visit. Kinematic measures were graphically represented as mean angular RMS amplitude and standard deviations of the population per joint over three trials per task and a log-transformation of the RMS datasets was applied for statistical analysis. Error bars representing standard deviations were excluded from plotted data to reduce graphical clutter. A linear mixed effects (LME) model via the maximized likelihood estimation was implemented using open-source statistical software, R (version 3.3.3). The effects of each clinical and kinematic outcome measures were examined for each participant group (ET and PD arms were separately analyzed thus no interaction effects were investigated) in separate LME models (“lme4” package using “lmer” function) [23]. Measures collected from pre-treatment (week 0) were compared to post-treatment visits (weeks 6, 16, 22, 32, 38, 48, 54, 64, 70, 80, 86, and 96). In addition, comparisons between peak effect of BoNT-A (across weeks 6, 22, 38, 54, 70 and 86), between re-injection visits (weeks 0, 16, 32, 48, 64, 80 and 96), and within each injection cycle (weeks 0-6-16, 16-22-32, 32-38-48, 48-54-64, 64-70-80, and 80-86-96) were analyzed. Each fixed effect was separately tested in a linear mixed effects model; fixed effects include: the mean angular joint tremor amplitudes for each scripted task (e.g. “load-1”), measured in RMS degree units and log-transformed for statistical testing, maximal grip strength, and clinical rating scales, such as the FTM rating scale, UPDRS scale, perceived weakness measured on the Likert scale, and quality of life scores (QUEST). LME allows adding research participants as a random effect to resolve issue of independence among repeated measures by controlling for individual variation among participants; the inclusion of subject as a random effect in the model assumes each participant has a unique intercept (baseline) for each outcome variable (e.g. UPDRS score, joint kinematic tremor severity per task). Estimated comparisons of least square means and 95% confidence intervals (95%CI) for each fixed effect in a LME model was calculated and multiple comparisons were adjusted using Tukey’s method (“lsmeans” package) [24]. An assessment of model fit was done by observing the normality of the errors via the residuals by observing the distribution of the residuals and the data was found to fit normality.

## Results

### Study demographics and BoNT-A treatments

Baseline clinical scores, demographics, and complete list of medications of the 24 ET and 28 PD participants have been previously published for this study [14,15]. Mean initial BoNT-A parameters were 174.1±68.8 units in 8.4±1.9 muscles for PD participants and 169.0±62.9 units in 8.8±2.0 muscles for ET participants [14,15]. While the average dose in both groups did increase by at least 30U during the initial dose optimization, the final PD and ET optimized dose did reduce to 180.3±74.8 units in 9.4±2.3 muscles and 188.5±78.1 units in 10.2±2.6 muscles, respectively (Tables 1 and 2).

**Table 1:** Optimization of BoNT-A parameters over the study treatment course for PD participants.

ID	Week 0 (First Injection)		Week 16 (Second Injection)			Week 32 (Third Injection)			Week 48 (Fourth Injection)			Week 64 (Fifth Injection)			Week 80 (Sixth Injection)		
	BoNT-A Dose (U)	No. of Muscles Injected	Event	BoNT-A Dose (U)	No. of Muscles Injected	Event	BoNT-A Dose (U)	No. of Muscles Injected	Event	BoNT-A Dose (U)	No. of Muscles Injected	Event	BoNT-A Dose (U)	No. of Muscles Injected	Event	BoNT-A Dose (U)	No. of Muscles Injected
**1**	100	7	G			D,M	75	8	D,D(M)	100	7	N	100	7	N	100	7
**2**	200	7	N	200	7	G			N	200	7	I,M	300	13	N	300	13
**3**	100	6	N	100	6	G			N	100	6	N	100	6	N	100	6
**4**	100	8	I	200	8	G			D	170	8	N	170	8	I	200	8
**5**	100	8	N	100	8	G			N	100	8	I,M	160	9	I	195	9
**6**	100	6	WD2														
**7**	200	8	G			N	200	8	N	200	8	N	200	8	G		
**8**	275	8	WD2														
**9**	260	9	I,M	390	11	G			WD3								
**10**	125	7	G			N	125	7	D	100	7	N	100	7	D	85	7
**11**	140	8	I,M	175	9	G			WD3								
**12**	100	8	I	170	8	D	100	8	I	170	8	N	170	8	N	170	8
**13**	175	8	N	175	8	D	135	8	I	175	8	N	175	8	WD4		
**14**	95	7	N	95	7	N	95	7	N	95	7	N	95	7	N	95	7
**15**	320	11	I,M	350	11	WD2											
**16**	200	11	WD1														
**17**	200	11	I,D(M)	280	9	I,D(M)	300	8	N	300	8	WD4					
**18**	200	10	N	200	10	N	200	10	N	200	10	N/A			N	200	10
**19**	200	6	WD2														
**20**	265	13	I	300	13	N	300	13	D	290	13	N	290	13	N	290	13
**21**	200	8	I,M	280	12	I,M	300	13	WD3								
**22**	200	8	D	100	8	N	100	8	N	100	8	N	100	8	N	100	8
**23**	190	11	D	100	11	N	170	11	N	170	11	N	170	11	N	170	11
**24**	200	8	I	200	8	M	200	11	N	200	11	N	200	11	N	200	11
**25**	300	12	D(M)	300	12	M	300	12	N	300	12	N	300	12	N	300	12
**26**	100	7	I,M	200	9	WD1											
**27**	130	9	I,M	200	11	N	200	11	N	200	11	N	200	11	N	200	11
**28**	100	6	D	80	6	WD4											
Mean	174.1	8.4		199.8	9.1		186.7	9.5		176.1	8.8		176.9	9.2		180.3	9.4
SD	68.0	1.9		89.2	2.0		82.3	2.1		69.5	2.0		70.9	2.3		74.8	2.3
Median	195.0	8.0		200.0	9.0		200.0	8.0		172.5	8.0		170.0	8.0		195.0	9.0
Range min	95.0	6.0		75.0	6.0		75.0	8.0		95.0	6.0		95.0	6.0		85.0	6.0
Range max	320.0	13.0		350.0	13.0		300.0	13.0		300.0	13.0		300.0	13.0		300.0	13.0

Abbreviations: M: Number of muscles increased; D(M): Number of muscles decreased; G: Participant had minimal tremor at visit and injector made clinical judgement to not inject; N: No change to parameters; D: decreased total dose; I: Increased total dose; N/A: Missed visit; WD^1^: Withdrawn due to lack of time commitment; WD^2^: Withdrawn due to unwanted weakness perceived my participant; WD^3^: Withdrawn due to participant perceiving no benefit; WD^4^: Withdrawn due to other health symptoms arose.

**Table 2:** Optimization of BoNT-A parameters over the study treatment course for ET participants.

ID	Week 0 (First Injection)		Week 16 (Second Injection)			Week 32 (Third Injection)			Week 48 (Fourth Injection)			Week 64 (Fifth Injection)			Week 80 (Sixth Injection)		
	BoNT-A Dose (U)	No. of Muscles Injected	Event	BoNT-A Dose (U)	No. of Muscles Injected	Event	BoNT-A Dose (U)	No. of Muscles Injected	Event	BoNT-A Dose (U)	No. of Muscles Injected	Event	BoNT-A Dose (U)	No. of Muscles Injected	Event	BoNT-A Dose (U)	No. of Muscles Injected
**1**	95	7	I,M	160	8	G			WD1								
**2**	100	6	I,M	200	13	G			D,D(M)	100	9	D,D(M)	85	7	N	85	7
**3**	160	8	I,M	290	13	N	290	13	N/A			N	290	13	N	290	13
**4**	70	4	I,M	200	8	G			N	200	8	WD2					
**5**	170	6	G			G			I,M	200	8	N	200	8	N	200	8
**6**	300	9	N	300	9	N	300	9	N	300	9	M	300	13	N	300	13
**7**	200	11	D	100	11	N	100	11	N	100	11	D	80	11	D,D(M)	70	9
**8**	200	9	D	150	9	N	150	9	N	150	9	N	150	9	N	150	9
**9**	195	9	I,M	300	12	D(M)	300	7	N	300	9	WD1					
**10**	185	10	N	185	10	I,M	200	13	I	255	13	N	255	13	N	255	13
**11**	100	8	I,M	200	11	N	200	11	N/A			N	200	11	WD3		
**12**	200	8	I,M	185	9	N	185	9	D	140	9	N/A			D,D(M)	100	7
**13**	170	10	N	170	10	D	165	10	I	190	10	N	190	10	N	190	10
**14**	200	11	I	260	11	I	300	11	WD3								
**15**	100	9	N/A			WD4											
**16**	200	10	N	200	10	I,M	260	13	I	280	13	N	280	13	N	280	13
**17**	300	11	M	300	14	WD2											
**18**	200	11	N	200	11	N	200	11	N	200	11	N	200	11	D	185	11
**19**	100	8	N	100	8	N	100	8	I	140	8	N	140	8	N	140	8
**20**	180	9	N	180	9	N	180	9	D	145	9	N	145	9	I,M	160	13
**21**	235	12	I	300	12	D	255	12	I	275	12	I,M	295	14	D	275	14
**22**	95	6	I	130	6	N	130	6	N	130	6	N	130	6	N	130	6
**23**	200	10	I,M	280	11	I	300	11	N	300	11	N	300	11	D	280	11
**24**	100	8	I,M	145	9	N	145	9	N	145	9	N	145	9	D	115	9
Mean	169.0	8.8		206.1	10.2		208.9	10.1		197.2	9.7		199.1	10.4		188.5	10.2
SD	62.9	2.0		65.8	1.9		71.0	2.0		71.0	1.8		75.5	2.4		78.1	2.6
Median	182.5	9.0		200.0	10.0		200.0	10.5		195.0	9.0		200.0	11.0		185.0	10.0
Range min	70.0	4.0		100.0	6.0		100.0	6.0		100.0	6.0		80.0	6.0		70.0	6.0
Range max	300.0	12.0		300.0	14.0		300.0	13.0		300.0	13.0		300.0	14.0		300.0	13.0

Abbreviations: M: Number of muscles increased; D(M): Number of muscles decreased; G: Participant had minimal tremor at visit and injector made clinical judgement to not inject; N: No change to parameters; D: decreased total dose; I: Increased total dose; N/A: Missed visit; WD^1^: Withdrawn due to lack of time commitment; WD^2^: Withdrawn due to unwanted weakness perceived my participant; WD^3^: Withdrawn due to participant perceiving no benefit; WD^4^: Withdrawn due to other health symptoms arose.

By week 80, 7% (2/28) PD participants withdrew from the study due to scheduling difficulties, 14% (4/28) withdrew due to bothersome hand weakness but had some tremor improvement, 11% (3/28) withdrew due to lack of benefit from treatment but did not have any weakness, and 11% (3/28) withdrew due changes in their health that were outside the study and could no longer participate (Table 1). For ET participants, 8% (2/24) withdrew due to scheduling conflicts, 8% (2/24) withdrew due to bothersome hand weakness but had tremor improvement, 8% (2/24) withdrew due to lack of improvement in tremor condition and without hand weakness, and 4% (1/24) withdrew due to other health concerns outside the scope of the study (Table 2).

### Tremor severity by UPDRS

**Fig 2:**
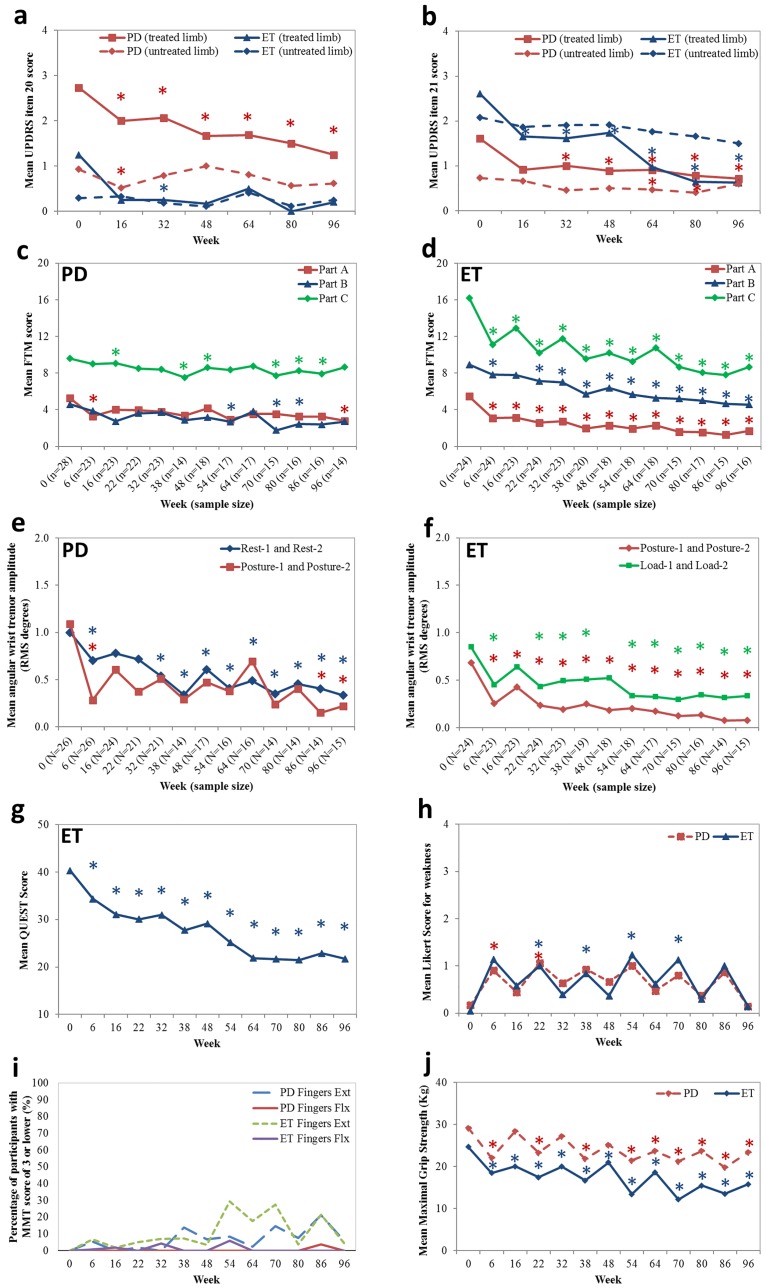
Significant effect of serial kinematically-based BoNT-A treatments on reducing tremor severity and functional disability caused by tremor and QoL improvements by validated clinical scales and kinematic tremor analysis along the whole-arm. (a-b) Mean UPDRS item 20 and 21 in the treated and untreated limbs in PD and ET participants; (c) Mean FTM part A-C scores in PD participants; (d) Mean FTM part A-C scores in ET participants; Mean angular RMS tremor amplitudes at the wrist in (e) PD participants and in (f) ET participants; (g) Mean QUEST score in ET participants; (h) mean Likert scale scores in PD and ET participants; (i) Percentage of participants who scored ≤3 on the MMT scale for finger flexion and extension, and (j) Mean maximal grip strength scores in the treated limb in both participant groups. Asterisks indicate statistical significance in means compared to week 0 and the asterisk colours are coordinated with each line plot (*). Injections were administered every 16 weeks starting at week 0.

Mean UPDRS items 20 and 21 scores, rest and action tremor respectively, for both limbs is displayed in Fig 2a and 2b. UPDRS ratings were conducted by a movement disorders neurologist who was blinded to previous ratings and the injected arm at the time of UPDRS assessment. A statistically significant reduction in rest tremor was observed in PD participants from week 0 to week 16 (*p*<0.001,95%CI -3.4,-1.0) and was maintained to week 96 (*p*<0.001,95%CI-5.0, -2.1). Rest tremor, detected in 8 of the 24 ET participants, was statistically significantly reduced from a mean rest tremor of 1.3±0.5 at week 0 to 0.2±0.4 UPDRS score at week 96 (*p* = 0.02,95%CI -4.8,-0.5). Action tremor in the treated limb (UPDRS item 21) was significantly reduced for PD participants from a mean score of 1.6±0.9 at week 0 to 0.7±0.7 at week 96 (*p* = 0.002,95%CI -3.7,-0.8). Mean UPDRS item 21 score in ET participants, was significantly reduced from 2.6±0.5 at week 0 to 0.6±0.7 UPDRS score at week 96 (*p*<0.0001,95%CI -6.2,-3.4).

Mean UPDRS item 20 for the untreated limb in PD participants was significantly reduced at week 16 (*p* = 0.02, 95%CI -3.2,-0.3), compared to baseline, and was not significantly different for the remainder of the treatment course (Fig 2a and 2b). Additionally, mean UPDRS item 21 for the untreated limb in PD was significantly reduced at week 64 (*p* = 0.04,95%CI -3.8,-0.1) and week 80 (*p* = 0.05,95%CI -4.2,-0.05), compared to baseline. Mean UPDRS item 21 score (action tremor) in the untreated limb for ET participants did not statistically significantly decrease over the treatment course, though there was a declining trend.

### Tremor severity and arm functionality

Mean FTM part A sub-scores for rest, postural and action tremors in the treated arm for PD and ET participants were plotted in Fig 2c and 2d, respectively. Mean FTM part A score in PD was statistically significantly reduced by a mean change of 36.6% at week 6 (*p* = 0.002,95%CI -0.7,-0.2) and of 46.3% at week 96 (*p* = 0.02,95%CI -0.5,-0.04). Mean FTM part A score in ET was significantly reduced from 5.5±1.9 at week 0 to 3.1±1.6 FTM points at week 6 *(p*<0.001,95%CI -8.0,-2.8) and was maintained throughout the treatment course to 1.7±1.1, a mean change of 66.1%, at week 96 (*p*<0.001,95%CI -4.4,-2.3); thus a qualitative reduction in tremor from a moderate-marked tremor at week 0 to a slightly perceivable to mild tremor at week 96.

Ability to pour liquids, and perform spiral and line drawing and writing tasks was assessed in all participants by FTM part B (Fig 2c and 2d). PD participants did significantly improve in the ability to write and pour liquids in their treated arm at week 54 (*p* = 0.04,95%CI -0.6,-0.02) which continued to week 80 (*p* = 0.03,95%CI -0.6,-0.02) and interestingly improvements were observed in their untreated arm at week 54 (*p* = 0.03,95%CI -0.6,-0.03) to week 96 (*p* = 0.02,95%CI -0.6,-0.1). ET participants did experience a significant improvement in the handwriting and pouring tasks from 8.9±3.4 at week 0 to 7.0±3.2 FTM points, a mean change of 36.4% at week 32 (*p* = 0.002,95%CI -4.0,-0.9) and was significantly maintained by a mean change of 44.9% up to week 96 (*p* = 0.02,95%CI -2.9,-0.2).

Functional disability caused by tremor was assessed by FTM part C and plotted in Fig 2c and 2d. Mean FTM part C score for eating, drinking, hygiene, and social activities in PD participants did significantly improve over the treatment course. Social activities significantly improved from 1.0±1.0 at week 0 to 0.8±1.2, a 12.9% change at week 16 (*p* = 0.03,95%CI -6.1,-0.3), where fine more tasks for the ability to eat without spills improved from 1.6±0.9 at week 0 to 1.1±0.7, a mean change of 34.8% at week 38 (*p* = 0.02,95%CI -4.3,-0.4) which was maintained by a mean change of 31.3% at week 86 (*p* = 0.03,95%CI -3.4,-0.2). ET participants benefited greatly from BoNT-A as the mean FTM part C score for eating, drinking, dressing, hygiene, work performance and social ability significantly improved from 16.2±4.6 at week 0 to 11.1±5.0 FTM points at week 6 (*p*<0.001,95%CI -7.6,-2.4) and continued to 8.7±4.3, a mean change of 46.3%, at week 96 (*p* = 0.03,95%CI -1.9,-0.1). As ET tremor was stabilized over the treatment course, functional benefits continued to improve with minimal functional interference by tremor.

### Kinematic analysis of tremor

Kinematics revealed a significant reduction in tremor in both PD and ET participants during all scripted tasks (Fig 2e and 2f). Mean PD wrist tremor captured during “rest-2” task was statistically significantly reduced from 1.3±1.1 RMS degrees at week 0 to 0.8±1.0 at week 6 (*p* = 0.04,95%CI -0.9,-0.02) and was maintained throughout treatment course to 0.3±0.3 RMS degrees, a mean change of 76.9%, at week 96 (*p* = 0.02,95%CI -0.8,-0.2) (Fig 2e). Similarly, mean wrist tremor during both postural tasks was significantly reduced by 72.7% from a mean tremor amplitude of 1.1±1.7 RMS at week 0 to 0.3±0.5 at week 6 (*p* = 0.01,95%CI -1.4,-0.2) and was stabilized over the study course to 0.2±0.3 RMS, a mean change of 81.8%, at week 96 (*p* = 0.03,95%CI -0.8,-0.04). Likewise, significant improvement in elbow tremor during rest and weight-bearing tasks were significantly improved at week 6 though this improvement was not maintained in the study course.

Mean wrist postural tremor in ET participants was significantly reduced by 61.6% at week 6 (*p*<0.001,95%CI -0.8,-0.3) and this tremor reduction was maintained at all subsequent time-points through to week 96 (*p*<0.001,95%CI -0.8,-0.3) when compared to baseline (Fig 2f). ET wrist tremor captured during weight-bearing tasks demonstrated a significant reduction in tremor amplitude at all time-points and was further significantly reduced following the fourth injection 0.6±0.6 RMS at week 48 to 0.3±0.3 RMS at week 64 (*p* = 0.004,95%CI -0.5,-0.1). Mean elbow tremor during postural tasks was significantly reduced by 37.0% from week 0 to week 6 (*p* = 0.003,95%CI -0.2,-0.03) and was further reduced by 40.7% from week 48 to week 64 (*p* = 0.004,95%CI -0.1,-0.001).

### Quality of life and muscle strength profile

Mean overall QoL rating, VAS score out of 100, reported by PD participants did not significantly change and remained statistically unchanged from the mean QoL score of 70.6±21.6 at week 0 throughout the treatment course (data not shown). Mean QUEST score for ET participants was significantly reduced from 40.3±15.8 QoL score at week 0 to 34.3±14.9 at week 6 (*p* = 0.002,95%CI -0.6,-0.1) and QoL continued to be significantly reduced by 46.1%, a score of 21.7±10.8 (*p*<0.001,95%CI -0.8,-0.4), at week 96 (Fig 2g).

A statistically significant increase in the mean Likert score for muscle weakness in injected muscles, perceived by PD participants, produced a value of 0.9±1.0 at week 6 (*p* = 0.009,95%CI 0.5,3.4) and 1.1±0.7 at week 22 (*p* = 0.03,95%CI 0.1,1.8) when compared to week 0 (no weakness), indicating mild perceived weakness following the first and second treatments, however this did not continue following week 22 (Fig 2h). ET participants also perceived weakness which was statistically significant from week 22 onwards. Perceived muscle weakness was not long-lasting over serial treatments as the mean Likert score was 0.1±0.4 at week 96 (*p* = 0.7,95%CI -0.9,1.3). Weakness rated by the Likert scale focused on treated muscles and excluded BoNT-A effects in non-injected muscles. The manual muscle testing (MMT) for finger flexors in the treated arm for both PD and ET participants showed minimum changes in strength over the course of 96 weeks (Fig 2i). At weeks 32, 54 and 86 less than 5% of all participants experienced finger flexor weakness with a score of 3 (fair) or lower. When assessing finger extensors, there was a presence of finger drop in the third, fourth and fifth digits with a MMT score lower than 3 in some PD and ET participants. For PD participants, only 5.2% (week 6), 13.8% (week 38), 6.6% (week 48), 8.2% (week 54), 14.6% (week 70), 7.5% (week 80), 21.3% (week 86) and 5.7% (week 96) of all measured fingers had scored less than or equal to 3 during MMT for finger extensors (Fig 2i). Likewise, for ET participants, 6.6% (week 6), 5.0% (week 22), 6.9% (week 32), 7.3% (week 38), 29.4% (week 54), 17.6% (week 64), 27.5% (week 70), and 21.4% (week 86) of all measured fingers scored 3 or less during MMT. Mean maximal grip strength was statistically significantly reduced following the first treatment and continued over the treatment course for ET participants (Fig 2j). Mean maximal grip strength was also significantly reduced at the six-week peak effect window following the first four treatments, however following the fifth treatment at week 64, mean maximal grip strength was significantly reduced at re-injection time-points in PD participants (Fig 2j).

A summary of all the means and standard deviations for all outcome measures across all study visits for PD and ET participants are tabulated in Tables 3 and 4 including *P* values < 0.05.

**Table 3:** Optimization of BoNT-A parameters over the study treatment course for PD participants.

	Week	0	6	16	22	32	38	48	54	64	70	80	86	96
**UPDRS 20**	**Untreated arm**	0.9 ± 1.1		0.5 ± 0.9 (0.02)		0.8 ± 1		1 ± 1.1		0.8 ± 1		0.6 ± 0.8		0.6 ± 0.8
	**Treated arm**	2.7 ± 0.6		2 ± 0.8 (<0.001)		2.1 ± 0.6 (0.002)		1.7 ± 1 (<0.001)		1.7 ± 1 (<0.001)		1.5 ± 1 (<0.001)		1.3 ± 0.9 (<0.001)
**UPDRS 21**	**Untreated arm**	0.7 ± 0.7		0.7 ± 0.7		0.5 ± 0.7		0.5 ± 0.8		0.5 ± 0.9 (0.04)		0.3 ± 0.7 (0.04)		0.5 ± 0.6
	**Treated arm**	1.6 ± 0.9		0.9 ± 1		1 ± 0.8 (0.02)		0.8 ± 0.9 (0.003)		1 ± 1 (0.004)		0.7 ± 0.9 (0.001)		0.8 ± 0.7 (0.002)
**FTM Part A Total**	**Untreated arm**	1.6 ± 1.7	1.8 ± 2	2.6 ± 3.3 (0.04)	1.8 ± 2.4	1.8 ± 2.3	2.8 ± 3	2.7 ± 2.8 (0.004)	1.7 ± 2.3	1.6 ± 2	1.6 ± 2.1	1.6 ± 1.9	1.9 ± 2.4	2.4 ± 2.9
	**Treated arm**	5.2 ± 2.1	3.3 ± 1.8 (0.002)	4 ± 2.3	4 ± 2.9	3.8 ± 2.8	3.4 ± 1.4	4.2 ± 2	2.9 ± 1.8	3.5 ± 2	3.5 ± 1.1	3.3 ± 1.5	3.3 ± 1.8	2.8 ± 1.7 (0.02)
**FTM Part B Total**	**Untreated arm**	3.8 ± 3.7	5.7 ± 5.2	3 ± 3.6	3.8 ± 4.1	3.5 ± 3.8	3.4 ± 3.9	3.4 ± 4.1	2.2 ± 2.3 (0.03)	4.1 ± 4.4	2.8 ± 3.7	2.9 ± 2.8	2.1 ± 2.5 (0.01)	2.4 ± 2.2 (0.02)
	**Treated arm**	4.6 ± 3.5	3.6 ± 3.1	3.8 ± 4.1	3.7 ± 3.6	3.8 ± 3.4	3.3 ± 2.8	3.4 ± 2.8	3.2 ± 2.4 (0.04)	3.8 ± 2.8	2.8 ± 2.2 (0.04)	2.9 ± 1.9 (0.03)	3.5 ± 2.5	3.5 ± 1.9
**FTM Part C Total**	**Eating**	1.6 ± 1	1.5 ± 1	1.5 ± 0.9	1.2 ± 0.9	1.4 ± 1	1.1 ± 0.7 (0.02)	1.3 ± 1.1 (0.02)	1.4 ± 1.2	1.2 ± 1	1.1 ± 0.9 (0.03)	1.1 ± 0.9 (0.01)	1.1 ± 1 (0.03)	1.3 ± 1
	**Drinking**	1.4 ± 1.1	1.2 ± 1	1.3 ± 0.9	1.2 ± 1	1.2 ± 0.8	1 ± 1	1.4 ± 1.4	1.4 ± 1.4	1.2 ± 1.1	0.9 ± 0.8	0.8 ± 0.7	1.2 ± 1	1.2 ± 0.6
	**Hygiene**	1.3 ± 1.2	1.3 ± 1	1.2 ± 0.9	1 ± 0.8	1 ± 0.9	0.9 ± 0.9 (0.05)	1.1 ± 1.1	0.9 ± 1	1.1 ± 1.1	1 ± 1	0.8 ± 0.9	0.9 ± 0.8	1 ± 0.7
	**Dressing**	1.3 ± 1.1	1.1 ± 1	1.3 ± 1.1	1.1 ± 0.9	1 ± 0.8	1 ± 0.7	1.2 ± 1.2	1 ± 1.2	1.3 ± 1.3	0.9 ± 1	1 ± 1	1 ± 1	0.8 ± 0.8
	**Working**	1.3 ± 1	1.3 ± 1.2	1.4 ± 0.9	1.3 ± 1	1.5 ± 0.9	1.1 ± 0.8	1.4 ± 1.1	1.1 ± 1.3	1.3 ± 0.8	1.4 ± 1	1.5 ± 0.9	1.1 ± 0.7	1.4 ± 0.9
	**Social Activities**	1 ± 1	0.8 ± 1	0.8 ± 1.2 (0.03)	0.9 ± 1.2	0.8 ± 1	0.7 ± 0.7	0.6 ± 1	0.6 ± 0.8	0.4 ± 0.6	0.4 ± 0.7	0.4 ± 0.7	0.4 ± 0.5	0.5 ± 0.5
	**Total**	9.6 ± 5.9	9 ± 5.7	9.1 ± 4.8 (0.03)	8.5 ± 4.2	8.4 ± 4.4	7.4 ± 4.1 (0.02)	8.6 ± 6.5 (0.02)	8.4 ± 6	8.8 ± 5.4	7.7 ± 4.9 (0.03)	8.3 ± 4.8 (0.01)	7.9 ± 4.3 (0.03)	8.6 ± 3.3
**QUEST**	**Total**	29.4 ± 15.1	30.6 ± 17.8	32.1 ± 17.9 (0.01)	27.3 ± 16.8	26.8 ± 17.1	27.6 ± 14.6	27.7 ± 15.9	28.3 ± 17.3	29.5 ± 17.8 (0.03)	22.7 ± 14.9	25.9 ± 15.4	24.7 ± 13.3	26.2 ± 14.7
**Maximal**	**Untreated arm**	32.3 ± 10.9	31.2 ± 9.7	33.1 ± 11.1	32.6 ± 10.8	34 ± 9.6	29.5 ± 10.4	29.9 ± 9.8	31.2 ± 9.8	28.9 ± 9.6 (0.04)	29.4 ± 9.2 (0.01)	30.3 ± 9.1 (0.007)	29.7 ± 9.6 (0.007)	29.1 ± 9.7 (0.003)
**Grip Strength**	**Treated arm**	29.2 ± 9.3	22.1 ± 7.7 (<0.001)	28.4 ± 8.6	23.3 ± 8.4 (0.03)	27.2 ± 8.2	21.9 ± 10.6 (0.01)	25.2 ± 7.9	21.5 ± 8.4 (0.002)	23.7 ± 8.6 (0.003)	21.3 ± 7.4 (0.01)	23.7 ± 8.4 (<0.001)	19.8 ± 7.8 (<0.001)	23.5 ± 9.8 (<0.001)
**Perceived weakness**	**Likert score**	0.4 ± 9.3	0.9 ± 7.7 (0.009)	0.6 ± 8.6	0.7 ± 8.4 (0.03)	0.7 ± 8.2	0.8 ± 10.6	0.9 ± 7.9	0.8 ± 8.4	0.6 ± 8.6	0.8 ± 7.4	0.6 ± 8.4	0.8 ± 7.8	0.4 ± 9.8

Summary table of clinical results for PD participants over the course of 96 weeks. All values represent mean with standard deviation with comparisons made to baseline.

**Table 4:** Optimization of BoNT-A parameters over the study treatment course for ET participants.

	Week	0	6	16	22	32	38	48	54	64	70	80	86	96
**UPDRS 20**	**Untreated arm**	0.3 ± 0.6		0.3 ± 0.8		0.2 ± 0.4		0.1 ± 0.3		0.4 ± 0.7		0.1 ± 0.3		0.3 ± 0.9
	**Treated arm**	1.3 ± 0.5		0.3 ± 0.7		0.3 ± 0.5 (0.02)		0.2 ± 0.4		0.5 ± 0.8		0 ± 0		0.2 ± 0.4
**UPDRS 21**	**Untreated arm**	2.1 ± 0.8		1.9 ± 0.8		1.9 ± 0.8		1.9 ± 0.9		1.8 ± 1		1.7 ± 1		1.5 ± 1
	**Treated arm**	2.6 ± 0.6		1.7 ± 0.9 (0.01)		1.6 ± 1.1 (<0.001)		1.7 ± 0.9 (0.003)		1 ± 0.6 (<0.001) *		0.7 ± 0.5 (<0.001)		0.8 ± 0.8 (0.001)
**FTM Part A Total**	**Untreated arm**	4 ± 2.4	3.4 ± 2.2	3.7 ± 2.1	3 ± 2.2	3 ± 2.3	2.7 ± 2.2	2.5 ± 1.8	2.9 ± 2.4	3.3 ± 2	3.4 ± 2.4	3.1 ± 1.7	2.8 ± 2.3	3.1 ± 1.9
	**Treated arm**	5.5 ± 1.9	3.1 ± 1.6 (<0.001)	3.1 ± 1.7 (<0.001)	2.6 ± 1.7 (<0.001)	2.7 ± 1.9 (<0.001)	2 ± 1.3 (<0.001)	2.3 ± 1.7 (<0.001)	1.9 ± 1.6 (<0.001)	2.3 ± 1.5 (<0.001)	1.6 ± 1.5 (<0.001)	1.5 ± 1.7 (<0.001)	1.3 ± 1.2 (<0.001)	1.7 ± 1.1 (<0.001)
**FTM Part B Total**	**Untreated arm**	9.2 ± 3.8	7.8 ± 3.6	8.1 ± 4.1	7.8 ± 3.6	7.9 ± 4	7.2 ± 3.5	7.1 ± 3	6.8 ± 3.3	6.7 ± 3.5	6.7 ± 3.4	6.2 ± 3	6.1 ± 3.7	6.1 ± 3.5
	**Treated arm**	8.9 ± 3.4	7.8 ± 3.8 (0.01)	7.8 ± 4.3	7.1 ± 3.5 (<0.001)	7 ± 3.2 (0.002)	5.7 ± 3.2 (0.005)	6.4 ± 3.9 (0.01)	5.7 ± 2.9 (0.01)	5.3 ± 2.3 (0.004)	5.2 ± 2.9 (0.004)	5 ± 2.6 (0.02)	4.7 ± 2.9 (0.02)	4.9 ± 2.2 (0.02)
**FTM Part C Total**	**Eating**	2.4 ± 0.9	1.8 ± 0.9 (0.005)	2 ± 0.8 (0.04)	1.6 ± 1 (0.001)	1.9 ± 1 (0.006)	1.5 ± 1.1 (0.002)	1.4 ± 1 (0.001)	1.4 ± 0.8 (<0.001)	1.8 ± 0.8 (0.005) *	1.5 ± 0.6 (0.001)	1.4 ± 0.6 (<0.001)	1.3 ± 0.5 (<0.001)	1.4 ± 0.8 (<0.001)
	**Drinking**	2.8 ± 0.8	1.9 ± 1.2	2.3 ± 0.9	2 ± 1.2 (0.03)	2.3 ± 1.2	1.7 ± 1.3	1.8 ± 1.3	1.5 ± 1.1 (0.04)	1.9 ± 1.1	1.5 ± 0.7 (0.03)	1.5 ± 0.9 (0.02)	1.1 ± 0.7 (<0.001)	1.3 ± 0.8 (<0.001)
	**Hygiene**	2.1 ± 1.2	1.1 ± 1 (<0.001)	1.4 ± 1.2 (0.004)	1.3 ± 1 (0.004)	1.3 ± 1.1 (0.005)	1.1 ± 0.9 (0.001)	1.4 ± 1 (0.03)	1.4 ± 1.1	1.3 ± 1.1	1.1 ± 1.1	0.9 ± 0.9 (0.04)	1.1 ± 1 (0.02)	1.1 ± 1 (0.03)
	**Dressing**	1.9 ± 0.9	1.4 ± 1	1.3 ± 1.1 (0.005)	1.2 ± 0.9 (0.002)	1.5 ± 1 (0.01)	1.2 ± 0.9 (0.004)	1.1 ± 1 (0.004)	1.3 ± 1 (0.01)	1.3 ± 1.1 (0.04)	1 ± 1.1 (0.03)	0.8 ± 0.8 (0.004) *	0.7 ± 0.9 (0.003)	0.9 ± 0.9 (0.004)
	**Working**	2.5 ± 1	1.5 ± 1 (<0.001)	1.7 ± 0.7 (0.001)	1.5 ± 0.9 (0.003)	1.6 ± 0.8 (0.005)	1.5 ± 1.1 (0.003)	1.7 ± 1 (0.03)	1.4 ± 1 (0.009)	1.4 ± 1.1 (0.03)	1 ± 0.8 (0.001)	1.1 ± 0.7 (<0.001)	1.3 ± 1 (<0.001)	1.5 ± 0.7 (<0.001)
	**Social Activities**	1.5 ± 1.3	0.8 ± 1.1 (0.005)	1 ± 1.1 (0.02)	0.5 ± 0.9 (<0.001)	0.7 ± 1.1 (0.002)	0.6 ± 1.1 (<0.001)	0.5 ± 0.7 (<0.001)	0.4 ± 0.7 (<0.001)	0.6 ± 0.8 (<0.001)	0.1 ± 0.4 (<0.001)	0.3 ± 0.6 (0.001)	0.1 ± 0.3 (<0.001)	0.2 ± 0.4 (<0.001)
	**Total**	16.2 ± 4.6	11.1 ± 5.0 (<0.001)	12.9 ± 6.0 (0.02)	10.2 ± 5.0 (0.03)	11.8 ± 5.3 (0.01)	9.6 ± 9.6 (0.004)	10.2 ± 5.2 (0.03)	9.3 ± 5.1 (0.04)	10.8 ±5.2 (0.04)	8.7 ± 2.7 (0.03)	8.1 ± 2.6 (0.04)	7.8 ± 3.5 (0.02)	8.7 ± 4.3 (0.03)
**QUEST**	**Total**	40.3 ± 15.8	34.3 ± 14.9 (0.002)	31.1 ± 15.3 (<0.001)	30 ± 15 (<0.001)	31 ± 15.8 (<0.001)	27.8 ± 15.3 (<0.001)	29.2 ± 14 (<0.001)	25.2 ± 15.1 (<0.001)	21.9 ± 10.9 (<0.001)	21.7 ± 9.2 (<0.001)	21.5 ± 9.3 (<0.001)	22.9 ± 12.8 (<0.001)	21.7 ± 10.8 (<0.001)
**Maximal Grip Strength**	**Untreated arm**	23.8 ± 11.1	24.3 ± 10.7	24.9 ± 9.8	22.1 ± 10.2	24.3 ± 10.2	23.1 ± 10	23.9 ± 10.4	19.5 ± 7.6	23.2 ± 10.1	21.3 ± 9.1	21.4 ± 9.2	24.4 ± 11	22.6 ± 9
	**Treated arm**	24.7 ± 10.7	18.5 ± 12.4	20.1 ± 10.1	17.4 ± 10.2	20 ± 10.7	16.7 ± 10	21 ± 10.7	13.5 ± 10.4 (0.02)	18.7 ± 10 (0.02)	12.2 ± 8.4 (0.03)	15.5 ± 8 (0.003) *	13.5 ± 8.4 (0.01)	15.8 ± 7.2 (<0.001)
**Perceived weakness**	**Likert score**	0 ± 0.2	1.1 ± 1.2	0.6 ± 1	1 ± 1 (0.003)	0.4 ± 0.7	0.8 ± 0.8 (0.04)	0.4 ± 0.5	1.2 ± 1 (0.01)	0.6 ± 0.9	1.1 ± 0.9 (0.02)	0.3 ± 0.6	1 ± 1.1	0.1 ± 0.4

Summary table of clinical results for ET participants over the course of 96 weeks. All values represent mean with standard deviation with comparisons made to baseline.

## Discussion

This is the first study to demonstrate the successful long-term treatment of PD and ET tremor over 96 weeks using BoNT-A (incobotulinumtoxinA). This longitudinal result after the initial reported 38 weeks shows continuous improvement in arm function in tremor patients with minimal risk of debilitating muscle weakness [14,15]. Using kinematic results to guide long-term optimization of BoNT-A dosing parameters for each patient, the outcome measures showed BoNT-A maintained a low and well-tolerated side effect profile in PD and ET participants and demonstrated significantly improved quality of life in ET participants. Kinematics enabled the ability to tailor BoNT-A to muscles that contribute to the overall tremor action at each arm joint. Objective measures of tremor following each serial treatment allowed optimization of BoNT-A therapy; adding/removing muscles and/or modifying dosages to ultimately improve outcomes and minimize any unwanted weakness or finger drop that could impair function. Long-term efficacy using BoNT-A is well known for disorders such as dystonia but not demonstrated in tremor therapy. This report shows that individualized, kinematically-guided BoNT-A treatment of ET and PD is safe and efficacious in the long-term as well as improved quality of life for ET patients [25,26].

Even though tremor is highly prevalent, disabling, and socially embarrassing, no new therapeutic options for tremor outside of surgery have been proposed in recent years. This significant stagnation in development of new therapies is reflected by a lack of investments by companies and institutes in the last four years [27]. The reluctance of physicians and patients to use pharmacological agents for treating tremor is a current concern due to the unpredictability of their therapeutic response and frequent side-effects [6,7,28]. The attraction to use a more targeted, localized therapy for tremor, like BoNT-A injections, has remained strong due to the focal nature of tremor. The difficulty in the visual assessment of multi-segment and multi-joint tremor has been a major road block in utilizing BoNT-A optimally. As clinical examination is commonly insufficient, kinematic analysis can measure and display accurate tremor amplitude readings aiding clinicians to correctly target muscles contributing to tremor in the arm, unique to each patient [28,29]. Rahimi and colleagues demonstrated that analyzing wrist tremor movements visually (clinical assessment by experienced injector) and objectively (using kinematics) produced a 36% and 53% agreement in muscle selection in ET and PD participants, respectively [30]. This demonstrated the low agreement between clinical and objective assessments and highlighted the inherent difference between these assessment techniques. Samotus and colleagues and Rahimi and colleagues reported effect of BoNT-A for ET and PD tremor, respectively, following a 38-week treatment period and strongly suggested that the use of kinematic tremor analysis could continue to better tailor BoNT-A parameters to changes in each individual’s tremor [14–16]. By using kinematic data instead of visual assessment, physicians that are expert injectors but not experienced tremor assessors have a better chance in treating this disorder with ease using this systematic approach. Thus, standardization of tremor measures and establishing injection parameters based on objective data can help improve the likelihood of successful outcomes.

Mean UPDRS scores, rating rest and action tremor, was significantly reduced by 34.9% following first treatment at week 16 and was further reduced by a mean change of 41.8% and 48.2% following the third and sixth treatments, respectively when compared to baseline. By week 96, PD tremor score for UPDRS was significantly decreased by 54.9% and FTM part A showed similar continuous reduction at week 6 and 96 (Fig 2a and 2c). Likewise, clinical FTM ratings of ET tremor significantly improved by 37.5%, 36.4%, and 50.9% at rest, action, and posture, respectively, following first treatment which was maintained up to week 96, resulting in a mean change in tremor severity by 55.0%, 63.6%, and 79.6% at rest, action, and posture, respectively (Fig 2d). This surprising trend suggests that with additional adjustment to BoNT-A over the two year period, tremor severity could continue to further decrease which was not captured in the previous papers [14,15].

Reductions in tremor, kinematically captured across all tasks, for PD and ET participants were measured every 6 and 16 weeks following a treatment visit. Kinematics revealed that the distribution of tremor at each joint (amount of tremor in each DOF contributing to the overall tremor) varies depending on the task or on the position of the arm. Yet by tailoring BoNT-A injections to the task which produced the highest tremor amplitude, tremor amplitude was significantly reduced for all tasks, and not for the individual task selected to tailor BoNT-A parameters/dosages. This suggests that tremor affects consistent muscle groups and that the severity of tremor from these muscles is modified by the relative voluntary activity or position of the arm joints. In addition, the severity of ET tremor during “posture-2” and “load-2” were significantly higher than the paired tasks, “posture-1” and “load-1”. These results also indicate that tremor severity is affected by the relative position of the arm, when semi-supinated at the forearm and flexed at the elbow while holding the weight of the object.

Both PD and ET participants who experienced functional disability caused by their tremor noticed significant improvement in performance of daily tasks such as eating solid foods and drinking liquids without spilling, completion of hobbies and an increase in socializing and dining out (Fig 2c and 2d). With such improvements, QoL was significantly improved for ET participants following the first treatment (Fig 2g). However, QoL was not statistically improved for PD participants as other motor and non-motor PD symptoms may have hindered QoL improvements. As expected, when BoNT-A wears off there was a predictable return in tremor severity on the day of re-treatment, however from our longitudinal results following week 48, it appears the peak return of kinematic tremor amplitude also continues to reduce (Fig 2e and 2f). This saw-tooth pattern appears to diminish with each serial treatment and was also recorded by the FTM scale. The functional improvements measured in FTM part B and C were immediate in the ET group at week 6 while the PD group seems to have a delayed yet more consistent improvement in arm function after week 48 (Tables 3 and 4).

During the 96 week treatment period only 14% (4/28) PD participants and 8% (2/24) ET participants withdrew due to bothersome weakness while 11% (3/28) PD participants and 8% (2/24) ET participants withdrew due to experiencing no functional benefit and no weakness. The remaining participants throughout this study did have functional benefit from treatment. In comparison, early tremor studies by Jankovic et al. (1996), 42–50% of patients reported mild hand weakness and 42% had moderate hand weakness with no functional hand improvement [12]. Similarly, in Brin et al. (2001), 30–70% of patients had reported adverse reactions relating to hand weakness when given 50-100U of BoNT-A with minimal functional improvement [31].

For future study design, a better rating scale for reporting changes in quality of life relating to tremor in PD participants should be considered since other Parkinson symptoms may have impacted the participant’s answers. While long term blinded studies of BoNT-A are not possible as muscle weakness can be easily recognized by patients and investigators resulting in immediate un-blinding, a lower dose using kinematic guidance should be explored to try and avoid this effect. Likewise, a blinded rater who is not determining the dosing parameter should also be considered in future studies to minimize any bias. Furthermore, literature is limited to studies reporting single injections or with booster injections, which has not demonstrated strong evidence that BoNT-A therapy can achieve both functional arm improvements with minimal muscle weakness. A recent review of tremor that was published prior to this study, stated the use of BoNT-A is supported by a level B and U recommendations (fixed dose of onabotulinumtoxinA and other BoNT-A formulations including incobotulinumtoxinA) and further evidence is needed [32]. The use of customized dosing parameters based on a patient’s tremor characteristic has been suggested [12], however, determining which muscles to target based on visual assessment remains to be extremely challenging. Therefore, the personalization of BoNT-A therapy using this kinematic approach provides each experienced and novice injector a user-friendly, standardized tool that is generalizable in the clinician’s hands.

Using kinematics, tremulous joints and the muscles contributing to debilitating tremor in ET and PD were objectively distinguished and targeted with BoNT-A. Following the first injections, a crucial contribution of kinematics is that it provides an objective measurement for determining adjustments to joint and muscle selection to guide BoNT-A dosing over serial treatments. Longitudinal and stable management of upper limb tremor by BoNT-A is therefore possible with such technology.

## Supporting information

S1 File The REB approved study protocol. (PDF)

S2 File The TREND statement checklist. (PDF)
